# Intratumoral Microbiome–Tumor Microenvironment Crosstalk in Breast Cancer Aggressiveness

**DOI:** 10.1155/tbj/1508684

**Published:** 2026-10-19

**Authors:** Oleksandr Mushii, Yelyzaveta Lukianova, Volodymyr Bazas

**Affiliations:** ^1^ RE Kavetsky Institute of Experimental Pathology, Oncology, and Radiobiology The NAS of Ukraine Zip Code: 03022, Vasylkivska Street, 45 Kyiv Ukraine; ^2^ Kyiv City Clinical Oncology Center Zip Code: 03115, Verhovynna Street, 69 Kyiv Ukraine

**Keywords:** breast cancer, intratumoral microbiome, microenvironment, tumor progression

## Abstract

Breast cancer (BC) remains the most common malignancy among women worldwide, with incidence and mortality rates continuing to rise. Alongside tumor heterogeneity and diverse clinical outcomes, increasing attention has been directed toward the role of the intratumoral microbiome as a regulator of breast tumorigenesis. Recent evidence demonstrates that breast tissue harbors one of the most diverse microbial communities compared with other cancer types, with marked differences between normobiota and malignant tissue. Intratumoral microorganisms are potentially associated with BC progression through multiple mechanisms, including modulation of inflammation, steroid hormone metabolism, and extracellular matrix remodeling. Specific taxa, including *Staphylococcus*, *Bacillus*, *Fusobacterium*, and *Paenibacillus*, have been associated with tumor aggressiveness, molecular subtypes, and patient prognosis. Moreover, the microbiome actively shapes the tumor microenvironment by altering immune infiltration, inducing resistance to anticancer therapy, and regulating immune checkpoint signaling, including PD‐1/PD‐L1 pathways. Microbial proteases and bacterial analogs of matrix metalloproteinases further contribute to extracellular matrix degradation and stromal remodeling, enhancing metastatic potential. Despite rapid advances, the clinical utility of intratumoral microbial signatures as predictive biomarkers of BC aggressiveness remains under investigation. Comprehensive characterization of microbial profiles across clinical and molecular subgroups may offer novel diagnostic, prognostic, and therapeutic opportunities in BC management.

## 1. Introduction

Statistical data from recent years indicate a steady increase in the incidence of breast cancer (BC). In terms of prevalence among all malignant neoplasms, BC ranks second (11.6%) after lung cancer; however, it holds absolute primacy among women. According to GLOBOCAN statistics, in 2022, 9.7 million people died from malignant neoplasms, with 6.9% of fatal cases attributed to BC (more than 650,000 deaths). At the same time, the projected increase in BC incidence over the next 25 years is estimated to range from 45% to 145% [1].

The decreasing average age of patients, the variability of tumor progression, and the pronounced heterogeneity of BC in terms of histological types and molecular subtypes highlight the need for a deeper understanding of the mechanisms underlying the pathogenesis of this cancer. In this context, in recent years, increasing attention has been directed toward studying the microbiota as a factor of systemic and local inflammation contributing to the development and progression of malignant neoplasms [2–4]. It has been demonstrated that microorganisms participate in the regulation of numerous physiological and pathological processes in the human body by influencing metabolic pathways and modulating signaling cascades.

Over the past few decades, significant progress has been made in understanding the interaction between the microbiome and the tumor site, and it has been established that the onset and progression of more than 20% of malignant neoplasms of different histogenesis are related to the procarcinogenic action of microflora [5]. Although the majority of studies in this field have focused on the influence of gastrointestinal microbiota, the discovery of a distinct intratumoral microbiome system has increasingly drawn the attention of researchers. This local microbial network plays a pivotal role in regulating tumor–host interactions [6]. Moreover, accumulating clinical evidence suggests that intratumoral conditionally pathogenic microbiota not only possess procarcinogenic properties but may also suppress the effectiveness of pharmacological treatment, contribute to the development of resistance to anticancer agents, and reduce the efficacy of certain immunotherapeutic approaches [4].

### 1.1. Tissue Normobiota of the Breast and Its Changes During Benign and Malignant Transformation

The development of molecular biology methods and the introduction of next‐generation sequencing technologies have, in recent years, made it possible to identify the biological diversity of intratumoral microbial populations in malignant neoplasms of different histogenesis, as well as to characterize their quantitative and qualitative features depending on the clinical status of cancer patients [6]. Notably, the greatest diversity of bacterial communities has been recorded in breast tissue, with a Shannon diversity index ranging from 0.8 to 5.2 and an average value of 3.8 (Figure 1). This level of diversity is comparable to that observed in oral and intestinal tissue samples, where diversity indices range from 3.9 to 6.5. These findings are most likely explained by the anatomical structure of the breast, which consists of adipose tissue with an extensive vascular network and lymphatic drainage, providing a favorable environment for bacterial growth. The mechanisms of breast microbiome formation remain incompletely understood. According to many researchers, bacterial migration may occur from the skin through the areola [7], while an alternative pathway involves the gut–mammary route, in which intestinal bacteria are transported to lymph nodes by immune cells and subsequently reach the bloodstream or lymphatic system [8].

**FIGURE 1:**
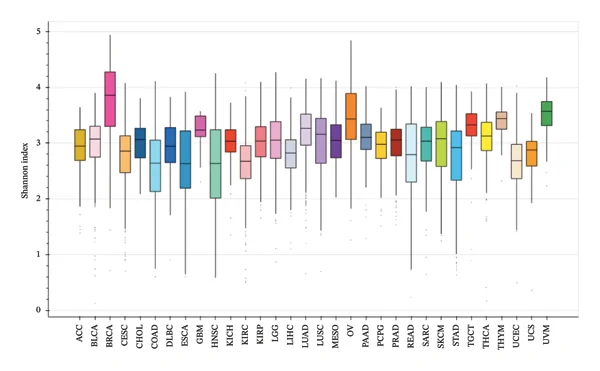
Shannon diversity index for the most common types of neoplasia, taxonomy level: genus (according to the bacteria in cancer (BIC) database; http://bic.jhlab.tw/).

Currently, it has been firmly established that the microbial composition of BC tissue is among the most diverse, both qualitatively and quantitatively, compared to malignant neoplasms of other localizations. On average, it contains 16.4 bacterial species compared to nine or fewer in other tumor types [9]. At the same time, the overall bacterial load in BC tissue is lower than in healthy breast tissue. In the breast tissue of conditionally healthy donors, bacteria of the genera *Lactococcus*, *Streptococcus*, *Prevotella*, *Corynebacterium*, and *Micrococcus* predominate. These genera are typical of the commensal microbiota of the skin, oral cavity, and mammary ducts and are considered relatively “peaceful” tissue colonizers. In contrast, BC tissue most often harbors bacteria of the genera *Staphylococcus*, *Bacillus*, *Lactobacillus*, *Pseudomonas*, *Neisseria*, *Fusobacterium*, and *Atopobium* [10, 11]. Importantly, intratumoral microbial representatives in BC are primarily intracellular and are localized within both transformed epithelial cells and immune cells.

The qualitative and quantitative composition of microbiota in normal and malignant breast tissue has not yet been fully clarified. Urbaniak et al. demonstrated that a distinctive feature of the intratumoral microbiota of BC is the high abundance of *Bacillus*, *Enterobacteriaceae*, and *Staphylococcus*. Furthermore, most *Bacillus* strains isolated from malignant breast tissue belonged to *Bacillus cereus* [12]. In another study [13], BC tissue exhibited an increased abundance of the *Proteobacteria* group, whereas in nontransformed tissue adjacent to the tumor, the relative amount of *Actinobacteria* was elevated.

According to Parhi et al. [10], *Fusobacterium nucleatum* was detected in 24% of BC tissue samples. The presence of this microorganism has previously been associated with an increased risk of colorectal cancer development and progression [11, 14]. However, its role within the intratumoral microbiota of BC remains unclear and requires further investigation.

Thus, in breast tissue of conditionally healthy donors, the predominant microbial representatives belong to the families *Streptococcaceae* (*Lactococcus*, *Streptococcus*), *Corynebacteriaceae*, *Micrococcaceae*, and *Methylobacteriaceae*. By contrast, in malignant breast tissue, there is an enrichment of intratumoral representatives from the families *Staphylococcaceae*, *Bacillacieae*, *Lactobacillaceae*, *Enterobacteriaceae*, *Comamonadaceae*, *Pseudomonadaceae*, *Dietziaceae*, *Genellaceae*, *Neisseriaceae*, *Fusobacteriaceae*, *Coriobacteriaceae* (*Atopobium*), *Acetobacteraceae*, and *Ralstoniaceae* (Figure 2).

**FIGURE 2:**
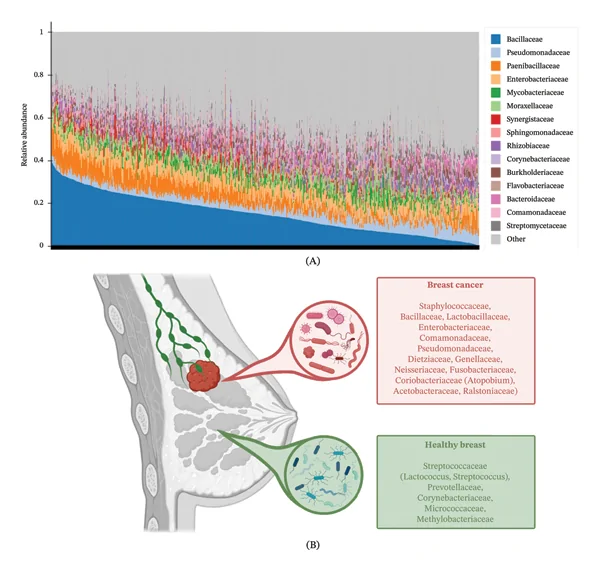
Mammary gland constitutive microbiome and intratumoral breast cancer microbiome composition: (A) top 15 families of microorganisms most represented in the intratumoral microbiome in BC (according to the Bacteria in Cancer (BIC) database); https://bic.jhlab.tw/; (B) typical representatives in the microbiome of healthy breast and BC tissue.

Such a profile looks like a transition from commensal to opportunistic‐pathobiont microbiota. This shift may reflect a transition from a protective commensal microbiota toward an opportunistic, inflammation‐prone microbial community. In addition, the formation of hypoxic conditions creates favorable conditions for the growth of opportunistic microbiota, while tumor‐induced immune suppression likely enables persistence of opportunistic bacteria that are normally cleared from healthy breast tissue. Moreover, the enrichment of metabolically versatile *Proteobacteria* and *Bacillus* species suggests that breast tumors may selectively favor bacteria capable of exploiting lactate‐rich, hypoxic, and nutrient‐limited environments. Collectively, available evidence supports the concept that BC is associated with a tumor‐driven microbial selection process. Hypoxia, metabolic reprogramming, immune suppression, and tissue remodeling create a niche that eliminates protective commensals while enriching metabolically adaptable, inflammation‐associated, and opportunistic bacteria.

### 1.2. Association of Intratumoral Microbiome Composition With Clinical Characteristics of BC

Recent research findings convincingly support the utility of detecting certain intratumoral microbiota members in the blood, as well as in biopsy [15] and surgical tissue samples [16], for predicting BC progression and informing treatment strategies (Table 1). Importantly, successful implementation of this approach in clinical practice requires a detailed characterization of age‐related changes in the intratumoral microbiota, as well as the identification of microbial profiles associated with the extent of BC dissemination.

**TABLE 1:** Changes in the composition of the intratumoral microbiome associated with the aggressiveness of the course of BC.

Clinical characteristics		BCa intratumoral microbiome alterations
*Patient-related factors*		
Age		Species & genus diversity with age↑

Postmenopause		Clostridiaceae↑, Oscillospiraceae (*Faecalibacterium*)↑, Ruminococcaceae↑ Lachnospiraceae (*Dorea*)↓, Lachnospiraceae↓

Survival rate	Low	Alcaligenaceae↑, Caulobacteraceae (*Brevundimonas*)↑, Enterobacteriaceae (*Proteus*)↑, Bradyrhizobiaceae↑
	High	Species & genus diversity in basal BCa↑ Species & genus diversity in ER‐positive tumors↓

*Tumor clinical characteristics*		
Stage	Low	Ruminococcaceae↑, Hyphomicrobiaceae↑
	High	Gemmatimonadaceae↑, Bradyrhizobiaceae (*Bosea*)↑, Mycoplasmoidaceae (*Tenericutes*)↑

Tumor size (T)	Smaller	Species & genus diversity↑
	Bigger	Oxalobacteraceae↑, Fusobacteriaceae (*Fusobacterium nucleatum*)↑

Lymph node involvement (N)	N1‐3	Pseudomonadaceae↑, Lactobacillaceae↑, Moraxellaceae (*Acinetobacter*)↑, Bacteroidaceae↑, Fusobacteriaceae (*Fusobacterium nucleatum*)↑ Moraxellaceae (*Alkanindiges*)↓, Alcaligenaceae (*Achromobacter*)↓

Distant metastasis (M)	M1	Staphylococcaceae↑, Caulobacteraceae (*Brevundimonas*)↑

Tumor grade (G)	Low	Microbacteriaceae (*Agrococcus*)↑ Bacteroidaceae↓
	High	Thermaceae (*Thermus*) ↓

*Tumor molecular characteristics*		
Molecular subtype	Luminal	Species & genus diversity ↑
	Her2/neu positive	Species & genus diversity ↑
	Basal	Pasteurellaceae (*Aggregatibacter*)↑, Streptococcaceae↑ Species & genus diversity ↓

ER status	Positive	Corynebacteriaceae↑, Tissierellaceae↑
	Negative	Actinomycetaceae↑, Chitinophagaceae↑, Methylobacteriaceae↑, Micrococcaceae↑, Moraxellaceae↑, Sphingomonadaceae↑, Xanthomonadaceae↑, Staphylococcaceae↑

HER2/neu status	Positive	Actinomycetaceae↑, Brucellaceae↑, Chitinophagaceae↑, Enterobacteriaceae↑, Methylobacteriaceae↑, Mycobacteriaceae↑, Streptococcaceae↑, Staphylococcaceae↑, Staphylococcaceae↑ Burkholderiaceae↓
	Negative	Flavobacteriaceae↑, Pseudoalteromonadaceae↑, Moraxellaceae↑

*Note:* ↑ increased abundance/diversity; ↓ decreased abundance/diversity.

It is known that the heterogeneity of breast microbiota composition is associated with patient age and menstrual status, likely due to the presence of chronic conditions and local inflammatory processes during aging [2], which lead to elevated levels of IL‐6, IL‐8, and TNF‐α [17]. These age‐related changes result in a reduction of tumor microbiota diversity in BC tissue and correlate with more aggressive tumor behavior [18]. Studies have demonstrated that postmenopausal BC patients exhibit higher levels of *Clostridiaceae*, *Faecalibacterium*, and *Ruminococcaceae*, alongside lower abundances of *Dorea* and *Lachnospiraceae*, compared with younger premenopausal patients [19]. Enrichment of estrobolome‐associated bacteria in postmenopausal patients may enhance enterohepatic estrogen recycling, thereby sustaining estrogen exposure despite ovarian senescence.

Furthermore, disease stage has been shown to correlate inversely with both the qualitative and quantitative composition of the intratumoral microbiome [20]. Specifically, stage I BC is characterized by an increased abundance of *Ruminococcaceae* and *Hyphomicrobium*, whereas tumors from stage II and III patients display enrichment of *Gemmatimonadetes*, *Tenericutes*, and *Bosea* [21]. Notably, the abundance of *Bosea* within the intratumoral microbiota correlates directly with the stage of basal‐like BC [22]. According to the results obtained using the Bacteria in Cancer (BIC, https://bic.jhlab.tw/) database, we demonstrated that the progression of BC stage positively correlates with the degree of microenvironmental infiltration by members of the family *Mycobacteriaceae* (*p* = 4.114*e* − 02) and the genus *Prevotella* (*p* = 7.044*e* − 04). Collectively, these findings suggest a stage‐dependent microbial succession in BC. Early tumors are associated with microbiota linked to metabolic homeostasis, whereas advanced stages display enrichment of stress‐resistant and inflammation‐associated bacteria. This shift may reflect increasing hypoxia, immune evasion, and chronic inflammation during tumor progression.

Literature data also indicate that tumor size is directly correlated with infiltration of BC tissue by members of *Oxalobacteraceae* [23] and *Fusobacterium nucleatum* [10]. In basal‐like BC, increasing tumor size is associated with a marked reduction in both qualitative and quantitative diversity of the intratumoral microbiota compared with T1 tumors [20].

Reports on the role of microorganisms in the lymphatic and hematogenous dissemination of BC are intriguing, although the available evidence remains limited and heterogeneous. Tzeng et al. demonstrated that lymphovascular invasion in BC tissue positively correlates with the abundance of *Lactobacillus*, *Acinetobacter*, and *Bacteroides*, while showing inverse correlations with *Alkanindiges* and *Achromobacter* [24]. Additionally, positive associations have been reported between the intratumoral abundance of *Fusobacterium nucleatum* [10] or *Pseudomonas aeruginosa* [23, 25] and the extent of regional lymph node involvement. However, these studies differ considerably in cohort composition, sample‐processing procedures, sequencing and analytical methods, and the taxonomic profiles identified. Consequently, the reported associations are not always directly comparable, and it remains unclear whether individual bacterial taxa actively promote metastatic dissemination or merely reflect changes in the tumor microenvironment associated with advanced disease.

Microorganisms residing within the tumor microenvironment may also be involved in distant metastasis. Experimental evidence obtained using the murine MMTV‐PyMT spontaneous breast tumor model suggests that intracellular bacteria may disseminate from the primary tumor within circulating tumor cells and promote pulmonary metastasis [26]. Chiba et al. also reported an increased intratumoral abundance of *Staphylococcus* and *Brevundimonas* in BC patients with distant metastases [25].

Mechanistically, microbiota associated with lymphovascular invasion may facilitate tumor‐cell intravasation through extracellular matrix (ECM) remodeling and the induction of epithelial–mesenchymal transition (EMT). Pro‐inflammatory bacteria may also enhance immune evasion and support tumor‐cell survival within lymphatic or blood vessels. Nevertheless, most clinical findings currently demonstrate associations rather than causality, while direct mechanistic evidence is derived predominantly from experimental models. Further standardized clinical and experimental studies are therefore required to determine whether the identified microorganisms are drivers, modifiers, or consequences of metastatic BC progression.

The highest intratumoral α‐diversity, which reflects the richness and distribution of microbial taxa within an individual tumor sample, alongside low intertumoral β‐diversity, which indicates relatively limited differences in microbial composition between tumor samples, has been observed in poorly differentiated G3 BC compared with highly differentiated G1 tumors [20, 27]. Poorly differentiated tumors are characterized by decreased abundance of *Bacteroidaceae* and increased abundance of *Agrococcus*. Additionally, bacterial DNA load was inversely related to disease status, and the expression of antimicrobial response genes in tumors was lower than in normal control tissue [27]. In another clinical observation, infiltration of BC tissue by *Thermus* has been positively correlated with tumor differentiation grade (*r* = 0.464, *p* = 0.029).

Recently, there has been growing interest in studying intra‐ and intertumoral heterogeneity of the microbiota across different molecular subtypes. This is particularly relevant because some microorganisms are involved in steroid hormone metabolism, secreting β‐glucuronidase and β‐glucosidase [28], which facilitate estrogen deconjugation and activate estrogen receptors [29]. In parallel, certain microbial strains, including *Bacillus cereus* [30], participate in progesterone receptor signaling by metabolizing progesterone to 5‐alpha‐pregnan‐3,20‐dione (5αP), thereby stimulating proliferation and metastatic potential in BC [31].

Nejman et al. demonstrated that hormone‐sensitive BC tissue shows a significant increase in the abundance of *Corynebacterium* and *Tissierellaceae*, whereas ER‐negative tumors display increased biodiversity among *Actinomycetaceae*, *Chitinophagaceae*, *Methylobacteriaceae*, *Micrococcaceae*, *Moraxellaceae*, *Sphingomonadaceae*, *Xanthomonadaceae*, *Staphylococcaceae*, and *Alkanindiges*. Meanwhile, members of *Streptococcaceae* were present with similar frequency in both hormone‐positive (*Streptococcus australis*) and hormone‐refractory (*Streptococcus cristatus*) BC. A notable shift toward *Flavobacteriaceae*, *Pseudoalteromonadaceae*, and *Moraxellaceae* within the intratumoral microbiome was observed in HER2/neu‐negative BC, whereas HER2/neu‐positive tumors exhibited higher abundances of *Actinomycetaceae*, *Brucellaceae*, *Chitinophagaceae*, *Enterobacteriaceae*, *Methylobacteriaceae*, *Mycobacteriaceae*, *Streptococcaceae*, and *Staphylococcaceae* [11]. Wang et al. reported a positive correlation between HER2 expression and intratumoral *Klebsiella* (*r* = 0.500, *p* = 0.017) and *Staphylococcus* (*r* = 0.462, *p* = 0.030), and an inverse correlation with *Burkholderia* (*r* = −0.469, *p* = 0.027) [23]. ER‐negative and HER2‐positive BC tumors were also characterized by broader species‐ and family‐level microbiota diversity [11]. Triple‐negative BC exhibited the lowest species diversity, enrichment of microorganisms from *Aggregatibacter*, *Plagiorchis*, and *Trichostrongylus*, and a significant increase in *Streptococcaceae* abundance within the intratumoral microbiome [20, 22].

Intratumoral microbiota appears to co‐evolve with BC molecular subtypes. Hormone‐dependent tumors may favor steroid‐metabolizing bacteria, HER2‐positive tumors enrich inflammation‐associated taxa, whereas aggressive hormone‐independent tumors exhibit increased microbial diversity or ecological collapse. These observations suggest bidirectional interactions between oncogenic signaling pathways and tumor‐associated microbial communities.

To date, the role of the intratumoral microbiota in predicting BC aggressiveness remains an open question. In basal‐like BC, longer relapse‐free survival was associated with lower microbial diversity in tumor tissue [20]. Conversely, ER‐positive tumors with higher microbial enrichment within the tumor microenvironment (TME) were associated with longer relapse‐free survival. Favorable prognosis in hormone‐sensitive BC correlated with higher infiltration of *Klebsiella* and *Stenotrophomonas*, whereas increased the relative abundance of *Alcaligenes*, *Brevundimonas*, and *Proteus* was associated with shorter relapse‐free survival [20].

In addition, using the BIC database, we found that among the 15 most represented genera in the BC microenvironment, *Paenibacillus* and *Burkholderia* exhibited the most promising prognostic significance. High infiltration of BC tissue by *Paenibacillus* was associated with a 10% decrease in overall 10‐year survival (*p* = 1.545*e* − 02). At the same time, patients with low levels of *Burkholderia* infiltration in BC tissue had a 25% lower 10‐year overall survival rate (*p* = 0.075); however, the difference compared to patients whose tumors exhibited higher levels of infiltration by this genus was not statistically significant.

Taken together, analysis of the existing literature on the relationship between intratumoral microbiota components, clinicopathological features, and patient survival indicates that high BC malignancy is associated with *Streptococcaceae*, *Staphylococcaceae*, *Bosea*, and *Fusobacterium nucleatum*.

### 1.3. Modulating Role of Intratumoral Microbiome in Immune and Stromal Components of the BC Microenvironment

Increasing evidence highlights the critical role of the intratumoral microbiome in modulating the biological properties of the breast TME at various stages of tumor development. Certain microbial species can stimulate inflammatory states and immune responses within breast tumor tissue, thereby promoting carcinogenesis and disease progression. Moreover, the diversity and composition of bacterial populations are associated with BC molecular profiles, contributing to the formation of a unique TME that facilitates tumor progression [32]. The mechanisms underlying interactions between microbiome members and TME components, as well as their regulatory factors, remain incompletely understood. Currently, the influence of the microbiome via reactive oxygen species (ROS) on the TME of malignancies, including BC, has been most extensively characterized [33].

Nevertheless, the impact of microbial members on immune and stromal cells within the tumor niche remains largely unresolved, and the causal effects of these interactions have not been fully identified. Some studies suggest that bacterial cells can penetrate immune cells and migrate with them to sites of inflammation [34]. Similarly, dissemination of bacteria alongside circulating metastatic tumor cells has been observed during metastasis. Fu et al. demonstrated that intracellular bacteria enhance resistance to fluid shear stress by inducing remodeling of the human cell actin cytoskeleton through RhoA inhibition and ROCK activation. This process increases metastatic cell survival during vascular transit, thereby augmenting metastatic potential [26].

Intratumoral microbiota may shape the immune phenotype of breast tumors. While some bacteria are associated with immune exclusion and poor prognosis, others correlate with enhanced infiltration of antitumor immune cells and improved survival. These findings suggest that tumor‐associated microbes may actively participate in determining immune‐hot and immune‐cold tumor states. Certain bacteria may contribute to immune exclusion by promoting chronic low‐grade inflammation and impairing antigen presentation. Shi et al. reported that the degree of lymphocytic infiltration in BC correlates with the β‐diversity of intratumoral bacteria. Tumors with low lymphocyte infiltration were enriched in *Mycobacterium*, *Rhodococcus*, *Catenibacterium*, *Bulleidia*, *Anaerofilum*, *Sneathia*, and *Devosia*, alongside low abundances of *Methanosphaera* and *Anaerobiospirillum* [35]. Some intratumoral bacteria may promote recruitment and activation of antitumor immune cells. A direct association was identified between lymphocytic infiltration and the abundance of *Clostridiales* (*r* = 0.452, *p* = 0.034), *Bacteroidales* (*r* = 0.496, *p* = 0.018), and *Azospirillum* (*r* = 0.431, *p* = 0.044), with an inverse relationship to *Streptophyta* content (*r* = −0.161, *p* = 0.024) [23].

Li et al., using TME‐based clustering of BC samples, showed that immune‐enriched tumors with high survival rates contained *Flammeovirga*, *Lachnoclostridium*, *Terrabacter*, *Gelidibacter*, *Pseudogulbenkiania*, and *Nitrospira*. The abundance of these microbial taxa positively correlated with infiltration of antitumor immune cells (NK cells, CD4+, and CD8+ T lymphocytes) and inversely with naive CD8+ T cells and monocytes. Conversely, immunodeficient tumor subtypes with low survival rates were characterized by intratumoral presence of *Simplexvirus*, *Pestivirus*, *Marichromatium*, *Pararhodospirillum*, *Acidibacillus*, *Candidatus Pelagibacter*, *Alcanivorax*, *Succinimonas*, *Oceanospirillum*, and *Roseivirga*, whose abundance negatively correlated with antitumor immune cell infiltration [36]. *Fusobacterium nucleatum* has been shown to suppress the activity of tumor‐infiltrating CD4+ and CD8+ T cells through activation of two immune checkpoint receptors, TIGIT and CEACAM1, thereby promoting tumor progression [37, 38]. The observed associations between intratumoral microbiota and lymphocytic infiltration suggest that tumor‐associated bacteria may actively shape immune‐hot and immune‐cold tumor phenotypes. Immunostimulatory taxa may enhance antigen presentation and T‐cell recruitment, whereas bacteria capable of inducing chronic low‐grade inflammation and intracellular persistence may promote immune tolerance and T‐cell exhaustion. Additionally, microbial biofilms and stromal localization may create physical and functional barriers that limit immune cell infiltration.

The SHIVA clinical trial revealed [18] that intratumoral microbiological diversity is associated not only with lymphocytic infiltration but also with PD‐L1 expression on immune and malignant cells, reflecting tumor aggressiveness [39]. Extracellular vesicles of bacterial origin play a key role in modulating immune checkpoint signaling, including PD‐1/PD‐L1 cascades [40]. Additionally, bacterial tryptophan metabolites, such as indoles, can modulate immune responses by inducing PD‐L1 expression and influencing CD8+ T cell activity via macrophage receptor interactions [41]. Certain bacterial consortia may enhance the efficacy of immune checkpoint inhibitors by stimulating dendritic cells to secrete IL‐12 and promoting cytotoxic T lymphocyte differentiation within tumors. Notably, Routy et al. reported that *Akkermansia muciniphila* improves anti‐PD‐1 therapy efficacy through an IL‐12‐dependent mechanism, enhancing recruitment of CCR9+CXCR3+CD4+ T cells to the TME [42].

Recent studies also highlight Toll‐like receptor (TLR)‐5‐mediated signaling through commensal bacteria recognition, leading to IL‐6‐driven inflammation and distant malignant transformation even in the absence of dysbiosis. Myeloid‐derived suppressor cells mobilized via IL‐6 induce γδ T cell production of galectin‐1, which suppresses antitumor immunity and accelerates tumor progression [43]. *Fusobacterium nucleatum* in tumor tissue can activate TLR4‐mediated signaling cascades, stimulating NF‐κB pathways and inducing proinflammatory and immune‐related gene expression [44]. Additionally, *Listeria monocytogenes* antigens have been shown to modulate myeloid‐derived suppressor cells, enhancing IL‐12 synthesis and promoting antigen‐specific adaptive immunity [45]. These alterations increase local tumor inflammation and autophagy, while inhibiting PI3K/Akt signaling [46, 47].

Collectively, these data indicate that intratumoral bacteria are actively involved in modulating the immune component of the BC microenvironment, primarily by promoting local and systemic inflammatory processes and disseminating via immune cells. Intratumoral microbiota play a key role in regulating functional activity of immune infiltrates, including macrophages and lymphocytes, through secretion of metabolites and extracellular vesicles, which suppress antitumor immunity and correlate with immunotherapy efficacy. A summarized scheme of intratumoral microbiome interactions with immune components of the BC microenvironment is presented in Figure 3.

**FIGURE 3:**
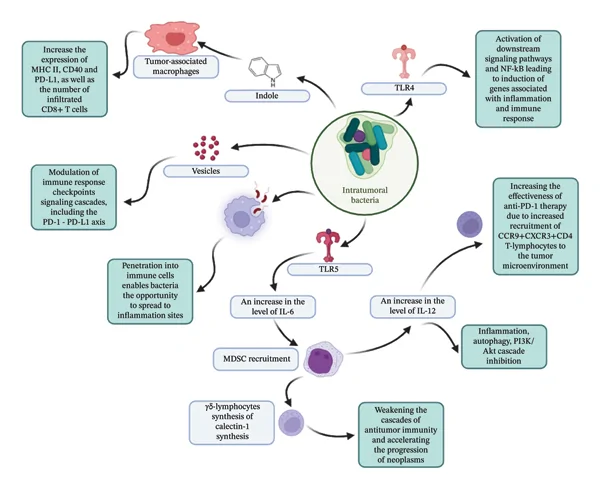
The influence of intratumoral microorganisms on the TME immune component of the BC. Created with BioRender.com.

In recent years, increasing attention has been paid to the influence of the microbiome on noncellular and proteinaceous components of the TME [20], which are involved in the formation of the three‐dimensional architecture of the BC ECM [48, 49]. Emerging studies suggest the potential use of fibrillar molecules within the tumor stroma as diagnostic markers for BC. Chekhun et al. [50] revealed that COL1A1 expression in BC tissue of young patients correlates with tumor aggressiveness and specific stromal features, indicating its potential diagnostic significance. Papanicolaou et al. [51] showed that collagen XII actively drives BC metastasis by orchestrating temporal changes in the TME and promoting matrix remodeling. Lukianova et al. [52] developed an algorithm for biomedical image analysis that enables quantitative assessment of the spatial collagen organization in BC tissue, highlighting the structural dimension of stromal biomarkers. Finally, Crespo‐Bravo et al. [53] demonstrated that serum type XII collagen is elevated in patients with solid tumors, reflecting fibroblast activation and offering a noninvasive marker of stromal remodeling.

Adipocyte‐rich tumor niches may provide a metabolically favorable and immunologically permissive environment for microbial colonization. Hieken et al. demonstrated a correlation between histological features of benign and malignant breast lesions and the presence of specific microbial taxa. In particular, infiltration of the TME by members of the *Firimicutes* and *Staphylococcaceae* families positively correlated with the proportion of adipose tissue and inversely with the content of the fibrillar ECM component in breast tumors [54]. The association of *Firmicutes* and *Staphylococcaceae* with adipose‐rich and collagen‐poor breast tumors suggests that intratumoral bacteria may participate in structural remodeling of the TME. Microbial proteases and metabolic activity may contribute to ECM degradation, adipose niche formation, and increased tumor invasiveness.

There is also evidence that elements of the intratumoral microbiota participate in modulating stromal remodeling molecules, such as matrix metalloproteinases (MMPs), as some microbes produce their own bacterial proteases [55, 56]. Most Gram‐positive bacteria—including *Streptococcus*, *Staphylococcus*, *Peptostreptococcus*, *Propionibacterium*, *Streptomyces*, and *Clostridium*—produce hyaluronidases to utilize hyaluronic acid as a carbon source and potentially facilitate pathogen dissemination through host mucosa [57]. These microbes are also capable of producing MMPs, particularly collagenases and gelatinases [58], and their bacterial analogs, which contribute directly to collagen matrix degradation, neoangiogenesis, and BC progression [59]. For example, in a mouse model of breast carcinoma, colonization with *E. coli* K‐12 markedly altered the TME by redistributing tumor‐associated macrophages and increasing TNF‐α and MMP‐9 expression [60]. Similarly, proteases and elastases from intratumoral *Pseudomonas aeruginosa* modulate local immune responses by degrading ECM components, inhibiting IL‐2‐induced lymphocyte proliferation, and inactivating IFN‐γ [61]. Mullberg et al. reported that bacterial proteases can mimic endogenous human membrane metalloproteinases, facilitating cleavage of extracellular domains of IL‐6 and TNF receptors [62]. Conversely, *Clostridium butyricum* MIYAIRI 588 within the BC microenvironment induces release of tumor necrosis factor–related apoptosis‐inducing ligand (TRAIL) from peripheral blood polymorphonuclear neutrophils, regulating the TLR2/4 signaling pathway via MMP‐8 [63].

Some evidence suggests that microbiota‐mediated modulation of stromal remodeling is linked to MMPs activity and microbial activation of mycothiol biosynthesis, which participates in ROS inactivation. Nejman et al. [11] demonstrated that distinct tumor types harbor specific intracellular bacteria that may influence stromal dynamics and metabolic adaptation within the TME. Consistent with this concept, Wang et al. [64] showed that mycothiol peroxidase (MPx) protects *Corynebacterium glutamicum* from acid‐induced oxidative stress by scavenging ROS, pointing to a bacterial mechanism of redox regulation. In BC cells, ROS‐dependent signaling is known to activate matrix‐degrading enzymes: Lee et al. [65] reported that tetrabromobisphenol A stimulates MMP‐9 expression through the NADPH oxidase–ROS–MAPK/Akt pathway. Similarly, Yang et al. [66] found that inhibition of NOX/ROS‐dependent NF‐κB signaling suppresses MMP‐9 and cell migration in astrocytes. In experimental BC models, ROS attenuation reduced collagen I production [67] and altered the redox equilibria of collagen‐functionalized nanomaterials relevant to cancer therapy [68]. Together, these findings suggest that microbiota‐related modulation of redox homeostasis and MMPs activity may critically shape stromal remodeling in the TME.

Beyond direct fibrillar matrix degradation, the microbiome may influence post‐translational modifications of collagen scaffold elements, altering their functional activity. For instance, *Porphyromonas gingivalis* is capable of citrullinating type I collagen, potentially affecting its interaction with fibroblasts via integrin α11β1 and promoting destructive tissue remodeling [69]. However, the role of microbiota‐induced post‐translational ECM modifications in BC initiation and progression remains incompletely understood.

Certain microbial metabolites also modulate EMT, thus promoting metastatic potential and cancer stem cell properties, contributing to BC progression. Specifically, indolepropionic acid, a tryptophan‐derived metabolite, can induce a reversion to the epithelial phenotype in BC cells by enhancing E‐cadherin expression [70]. Analysis of 668 BC tissue samples from The Cancer Genome Atlas revealed correlations between microbiome composition and gene expression profiles in tumor cells, with *Listeria fleischmannii* infiltration associated with altered expression of EMT‐related genes [13]. Experimental studies confirmed that quercetin, a microbial metabolite present in BC tissue, inhibits EMT, reduces invasiveness, and diminishes stemness by decreasing mitochondrial oxidative processes [71]. Additionally, *Streptococcus pyogenes* produces streptolysin O [72], which activates the EGF receptor ErbB1, reducing BC cell invasiveness in synthetic ECM models [73].

Taken together, these findings indicate that intratumoral microbes directly contribute to shaping the stromal architecture of BC. Bacterial units can modulate the assembly of the three‐dimensional collagen network and secrete their own endopeptidases homologous to eukaryotic MMPs and their inhibitors. Moreover, the microbiota participates in regulating ROS formation, indirectly influencing the activity of stromal remodeling proteins within BC tumors. A summarized scheme illustrating the impact of intratumoral microorganisms on the stromal components of the TME is shown in Figure 4.

**FIGURE 4:**
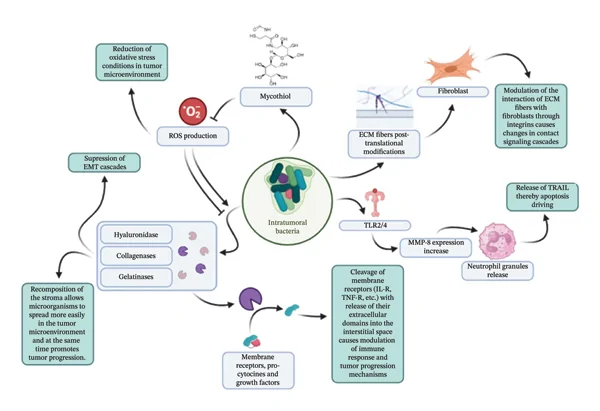
The impact of intratumoral microorganisms on TME noncellular components and protein elements of the BC. Created with BioRender.com.

To explore functional processes associated with the BC intratumoral microbiome, we used the BIC database [74], which provides built‐in functional analysis and visualization tools. Five microbial families most abundantly represented in the BC TME were selected based on the database summary statistics. Functional associations with Gene Ontology Biological Processes, KEGG pathways, and Reactome pathways were explored using the BIC enrichment visualization interface. All enrichment scores and statistical procedures were generated internally by the BIC platform; no additional independent enrichment calculations were performed in this study. Our findings revealed that members of the families *Pseudomonadaceae*, *Enterobacteriaceae*, and *Mycobacteriaceae* are directly involved in modulating cytokine profile formation, regulating inflammatory processes, and shaping immune responses within the BC TME. At the same time, representatives of all five examined families were associated with processes related to ECM remodeling in breast tumors. Notably, we also identified the involvement of *Pseudomonadaceae*, *Paenibacillaceae*, and *Enterobacteriaceae* in the regulation of tumor vascular network formation (Figure 5).

**FIGURE 5:**
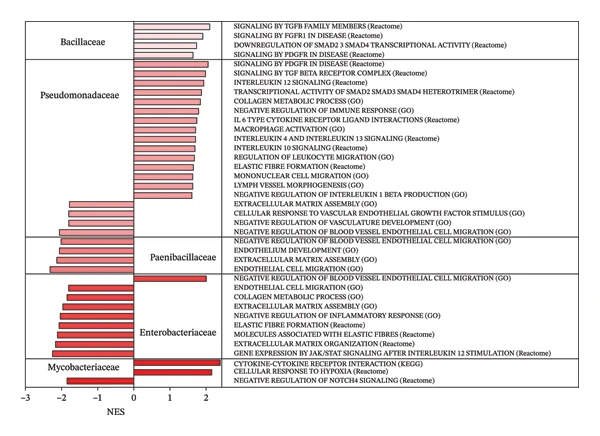
Bacteria‐associated biological functions of the top 5 most represented bacterial families in the BC microenvironment. Analysis was performed using the bacteria in cancer database (BIC, http://bic.jhlab.tw/). The figure shows only those biological processes and signaling pathways that are associated with the BC microenvironment, demonstrate sufficient enrichment (NES > 1.8), and are statistically significant (adjusted *p* < 0.05). NES—normalized enrichment score, a normalized measure of enrichment that reflects the degree of enrichment independently of the gene set size.

### 1.4. Translational Potential of Intratumoral Microbiome in the Era of Personalized Medicine

As mentioned above, resident microorganisms of BC tissue are capable of modulating tumor progression indirectly through the activity of their own metabolites. However, the involvement of similar metabolic pathways in modulating therapeutic effects during the treatment of this type of neoplasm should also be considered. It has been demonstrated that bacteria within the TME can metabolize the chemotherapeutic drug gemcitabine (2′,2′‐difluorodeoxycytidine) into its inactive form, 2′,2′‐difluorodeoxyuridine. This metabolism depends on the expression of the long isoform of the bacterial enzyme cytidine deaminase, which is mainly characteristic of Gammaproteobacteria. In an in vivo model, resistance to gemcitabine was induced by intratumoral Gammaproteobacteria and was subsequently overcome by treatment with the antibiotic ciprofloxacin [75]. It has also been shown that culturing cells in the presence of doxorubicin together with *E. coli* led to attenuation of the drug’s cytotoxic effect compared with the control without microorganisms [76].

An association between the presence of specific microbial genera within the TME and therapy efficacy in BC has also been reported. Tumor infiltration by metabolism‐associated genera, particularly *Campylobacter* and *Nitrosopelagicus*, was inversely correlated with drug sensitivity to zoledronate, alpelisib, fulvestrant, and nelarabine [77]. *Lachnoclostridium* showed a positive association with the IC50 of tamoxifen, docetaxel, sorafenib, cetuximab, and veliparib, but a negative association with the IC50 of sunitinib, methotrexate, bleomycin, and gemcitabine [36]. A univariate survival analysis of metabolism‐associated microorganisms performed by Chen et al. demonstrated that *Campylobacter*, *Saccharibacter*, *Paludibacter*, *Lawsonia*, and *Bulleidia* were prognostic factors in patients with BC. Moreover, the authors developed a risk‐score model based on the composition of the intratumoral microbiota: Risk score = (Campylobacter × 0.328) + (Paludibacter × 0.285) + (Marichromatium × −0.194). The 10‐year AUC value of the model was 0.662, indicating accurate long‐term prognostic performance [77].

Interesting findings by Geng et al. demonstrated the formation of an immunosuppressive BC TME under the influence of the commensal microorganism *Fusobacterium nucleatum*. The authors showed that reversible changes in the microenvironment could be achieved using antibiotic agents, resulting in improved response to immune checkpoint blockade therapy [78]. At the same time, the use of kefir‐based probiotics demonstrated similar effects: cells cultured with probiotic agents exhibited reduced concentrations of pro‐inflammatory and pro‐angiogenic factors in the culture medium [79].

The reverse effects of therapy on the qualitative and quantitative composition of the intratumoral microbiota in breast tumors should also be considered. Clinical studies indicate that after neoadjuvant polychemotherapy, the abundance of *Pseudomonas* increased, whereas *Prevotella* decreased, although these changes did not affect clinical outcomes [25].

The rapid development of nanotechnology and bioengineering now makes it possible to modify natural bacteria according to specific therapeutic needs. The main effects and advantages of treatment using such modified microorganisms include the ability to influence apoptosis and autophagy in transformed cells, modulate immune responses and metabolic processes in tumor cells, serve as delivery vehicles for therapeutic agents, or directly produce such compounds at the site of tumor infiltration. A related research direction involves the use of viral agents for similar purposes [80].

A novel interdisciplinary field at the interface of oncology and microbiology focuses on the search for tumor‐lytic microbial strains capable of exerting suppressive effects on tumors and possessing therapeutic potential. Goto et al. published a series of studies demonstrating the potential use of *Rhodopseudomonas palustris* and *Proteus mirabilis* in the treatment of tumors of various origins. Chemotherapy‐resistant BC tumors in mice (derived from the EMT6/AR1 line) showed a positive clinical response after administration of a bacterial consortium of these species. A repeated single intravenous injection of the combination of *Rhodopseudomonas palustris* and *Proteus mirabilis* improved survival in model animals. The authors reported that the use of this microbial combination as a therapeutic agent was safe and did not cause adverse effects during treatment [81]. In addition, *Rhodopseudomonas palustris* exhibits NIR‐I‐to‐NIR‐II reporter fluorescence [82] and can be used for diagnostic purposes due to its ability to specifically colonize tumor tissue [83]. Loeffler et al. genetically engineered a strain of *Salmonella typhimurium* capable of expressing Fas ligand, involved in apoptosis activation. This approach reduced BC tumor size in animal models by 60% and decreased metastasis frequency by 32% [84].

## 2. Conclusions

In summary, current literature indicates that the intratumoral microbiome represents a crucial yet underexplored component of the BC TME. The presence and activity of microorganisms within tumor tissue extend far beyond simple colonization; they actively influence key cellular and molecular mechanisms that govern tumor initiation and progression.

The intratumoral microbiota represents an active participant in BC biology rather than a passive colonizer. Age‐ and menopausal status–related changes reduce microbial diversity and promote a pro‐inflammatory milieu (IL‐6, IL‐8, TNF‐α), facilitating aggressive tumor behavior. Specific taxa correlate with tumor stage, size, grade, and molecular subtype: e.g., enrichment of *Fusobacterium nucleatum*, *Pseudomonas*, and *Bosea* associates with higher stage and nodal involvement, whereas distinct microbial profiles characterize ER+, HER2+, and triple‐negative BC.

Microorganisms contribute to BC progression by modulating hormone metabolism (β‐glucuronidase/β‐glucosidase‐mediated estrogen reactivation, progesterone metabolites), remodeling the tumor immune microenvironment (promotion of immunosuppressive cells, lymphovascular invasion), and supporting metastatic dissemination, including via intracellular carriage within circulating tumor cells. Microbial dysbiosis also reflects differentiation status and impacts patient prognosis: higher enrichment of *Klebsiella* and *Stenotrophomonas* is favorable, whereas *Alcaligenes*, *Brevundimonas*, and *Proteus* associate with poor survival.

Notably, several microbial species—including *Fusobacterium nucleatum*, *Listeria monocytogenes*, *Pseudomonas aeruginosa*, *Clostridium butyricum*, and *Porphyromonas gingivalis*—are not only associated with malignant transformation but also directly modulate the state of the TME.

These bacteria can affect immune cells by either promoting or suppressing antitumor responses, thereby facilitating immune evasion by tumor cells. They also regulate local inflammatory reactions, which, when chronic, create a permissive environment for tumor growth and invasion. Additionally, the microbiome plays a pivotal role in remodeling the ECM and altering the architectural organization of the TME, directly impacting the invasive and metastatic potential of BC cells.

Emerging evidence highlights that the microbiome and its molecular components represent not only a critical part of the TME but also a promising avenue for therapeutic intervention. Modulation of microbial communities and the use of their bioactive products could potentially reshape immune responses, influence tumor progression, and enhance the efficacy of existing anticancer strategies. Results of our previous study [85] demonstrated that a lectin derived from *Bacillus subtilis* IMV B‐7724 can modulate PD‐L1 expression in BC cells of varying malignancy, suggesting that bacterial bioactive molecules may influence tumor immune evasion mechanisms. Expanding this concept, Wang et al. [86] comprehensively reviewed how the intratumoral microbiome contributes to immune modulation and opens new perspectives for innovative therapeutic strategies, including microbiota‐based immunomodulation and metabolic intervention in cancer.

Although this field remains at an early stage, it opens an important direction for future research aimed at integrating microbiome‐based approaches into the management of BC. Thus, the tumor microbiome should not be viewed merely as a passive component of cancerous tissue. Rather, it acts as an active regulator, shaping the balance between antitumor and pro‐oncogenic signals and determining tumor aggressiveness and malignancy. Further in‐depth investigation of the intratumoral microbiome holds promise for the development of novel diagnostic and prognostic biomarkers and may provide a foundation for innovative therapeutic strategies aimed at modulating the microbial composition of tumors or blocking its pathological effects.

**Nomenclature**
BC: Breast cancer
BIC: Bacteria in Cancer (database)
ECM: Extracellular matrix
EMT: Epithelial–mesenchymal transition
ER: Estrogen receptor
GLOBOCAN: Global cancer observatory
HER2: Human epidermal growth factor receptor 2
IFN‐γ: Interferon gamma
IL: Interleukin
MMP: Matrix metalloproteinase
NK cells: Natural killer cells
PD‐1: Programmed cell death protein 1
PD‐L1: Programmed death‐ligand 1
ROS: Reactive oxygen species
TIGIT: T cell immunoreceptor with Ig and ITIM domains
TLR: Toll‐like receptor
TME: Tumor microenvironment
TNF‐α: Tumor necrosis factor alpha
TRAIL: Tumor necrosis factor–related apoptosis‐inducing ligand

## Funding

The study was conducted within the framework of the Institute of RE Kavetsky Institute of Experimental Pathology, Oncology, and Radiobiology’s routine research activities, without additional external funding.

## Conflicts of Interest

The authors declare no conflicts of interest.

## Data Availability

The data that support the findings of this study are available from the corresponding author upon reasonable request.
